# Evaluation of colistin stability in agar and comparison of four methods for MIC testing of colistin

**DOI:** 10.1007/s10096-017-3140-3

**Published:** 2017-11-25

**Authors:** Agata Turlej-Rogacka, Basil Britto Xavier, Lore Janssens, Christine Lammens, Olympia Zarkotou, Spyros Pournaras, Herman Goossens, Surbhi Malhotra-Kumar

**Affiliations:** 1 Laboratory of Medical Microbiology, Vaccine & Infectious Disease Institute, University of Antwerp, Antwerp, Belgium; 2 Department of Microbiology, Medical School, University of Athens, Athens, Greece; 3 Department of Medical Microbiology, Campus Drie Eiken, University of Antwerp, S6, Universiteitsplein 1, B-2610 Wilrijk, Belgium

**Keywords:** Polymyxins, Susceptibility testing, Stability, Reproducibility, Agar dilution, E-test, Etest, Broth microdilution, Broth macrodilution, Heteroresistance, Skip wells

## Abstract

Susceptibility testing for colistin remains challenging primarily due to its inherent properties. We evaluated colistin stability in agar and reproducibility of colistin MICs obtained by agar dilution, broth macro- and micro-dilution and MIC gradient strips on 3–7 iterations of each method using clinical *Klebsiella pneumoniae* (susceptible-CS, and resistant-CR, *n* = 2 each), *mcr-*harboring *Escherichia coli* (n = 2), and reference strains *E. coli* ATCC25922 and *Pseudomonas aeruginosa* ATCC27853. MICs for reference strains were not in the given range using Etest and broth microdilution (ATCC25922, 0.125 and 4 μg/ml, respectively). MICs of CR-1 and CR-2, and of the *mcr-*harboring *E. coli* showed high concordance between agar and broth dilution varying up to one 2-fold dilution. However, remarkable variations were observed on broth dilution with CS-1 and CS-2 (MIC range 0.25–32 and 0.5–64 μg/ml, respectively); whereas for agar dilution the MIC for both CS strains was 0.5 μg/ml in all the runs. MICs obtained by MIC gradient strips were lower than those obtained by dilution methods (1–2 dilutions for CS and *mcr* strains, and up to five dilutions for CR strains). To confirm uniform distribution of colistin in agar, a single strain was spotted in five different regions of the same plate. All spots showed concordant growth with maximum one dilution difference. No effect on MIC was found due to storage of colistin-containing agar plates for 7 days at 4 °C. In our hands, agar dilution was superior in terms of reproducibility and robustness, compared to broth dilution methods, for colistin MIC determination.

## Introduction

Emergence of multi-drug resistance among clinically important Gram-negative bacteria has facilitated the re-introduction of old antibiotics, such as colistin, into clinical use [1]. Expectedly, resistance to colistin has also emerged in Gram-negative pathogens such as *Acinetobacter spp.*, *Pseudomonas aeruginosa*, *Escherichia coli* and *Klebsiella spp*. [2], mediated by chromosomal mutations as well as by genes such as *mcr-1* and *mcr-2* present on mobile elements [3, 4]. This underscores the urgent need for standardized in vitro susceptibility testing by clinical microbiology laboratories both for patient care and for epidemiological surveillance. However, this has been a challenging task because of the inherent properties of colistin such as its cationic nature, an affinity for plastic as well as a poor diffusibility in agar [5, 6].

Poor diffusion in agar negates the use of the disk diffusion technique, which was found unreliable by several studies [7–11], and also of MIC testing with the gradient strip method [12]. Although, some studies have also shown very good agreement between colistin gradient strip and agar dilution MIC methods [7, 13, 14]. Another commonly employed method is broth dilution method; however, binding of colistin to plastic remains an issue. The use of a surfactant (Polysorbate 80) to limit the absorption to plastic was suggested [6]; however, this had to be abandoned due to an observed synergistic effect of Polysorbate 80 with colistin [12]. A common approach to overcome binding to plastic has been to perform MIC testing in glass tubes (broth macrodilution). It is, however, very difficult to avoid any contact with plastic during the entire procedure and this method is also very labor-intensive, limiting its utility to research-based endeavors.

Adding further complexity to MIC testing is the as-yet poorly understood phenomenon of heteroresistance, which essentially refers to the presence of a subpopulation of colistin-resistant bacteria within an apparently susceptible bacterial population [7, 15].

Currently, the joint CLSI-EUCAST Polymyxin Breakpoints Working Group recommends the use of broth microdilution in plain polystyrene trays using cation-adjusted Mueller-Hinton broth without any additives (http://www.eucast.org/ast_of_bacteria/guidance_documents), but also states the need for further investigations of agar dilution MIC determination. Because of the growing importance and urgent need to define an optimal, user-friendly method for susceptibility testing for colistin, we evaluated agar plates for evenness of colistin distribution and assessed its stability over one week in comparison to currently utilized colistin MIC testing methods, i.e., broth macrodilution, broth microdilution, gradient MIC strip and agar dilution.

## Materials and methods

### Bacterial strains

Four clinical *Klebsiella pneumoniae*, colistin-susceptible (CS-1 and CS-2) and -resistant (CR-1 and CR-2), as well as *mcr-1*- and *mcr-*2- harboring *E. coli* (*n* = 2) were studied. CS-1 and CS-2 exhibited colistin MICs of 0.5–1 μg/ml and 1–2 μg/ml, respectively, CR-1 and CR-2 of 64 μg/ml, and *mcr-1* and *mcr-2* of 4–8 μg/ml by broth macrodilution. The two control strains, ATCC 25922 *E. coli* and ATCC 27853 *P. aeruginosa*, were obtained from ATCC collection (Table 1).

**Table 1:** Current CLSI and EUCAST MIC breakpoints for colistin

Genera	CLSI breakpoints (μl/ml)			EUCAST breakpoints (μl/ml)		CLSI recommended quality control strains	
	S	I	R	S	R	Strain	MIC (μl/ml)
Enterobacteriaceae	≤2	–	≥4	≤2	>2	ATCC 25922 (*E. coli*)	0.25–2
Pseudomonas	≤2	–	≥4	≤2	>2	ATCC 27853 (*P. aeruginosa*)	0.5–4

*CLSI* Clinical and Laboratory Standards Institute, *EUCAST* European Committee on Antimicrobial Susceptibility Testing, *MIC* minimum inhibitory concentration, *S* susceptible, *I* intermediate, *R* resistant

### Study design

We investigated broth macrodilution, broth microdilution, MIC gradient strips and agar dilution for colistin MIC testing in terms of reproducibility and inter-investigator variability. Overall, reproducibility was tested at least in three independent experiments for each strain from a freshly prepared inoculum except the agar dilution method with CS and CR strains that was performed in duplicate (plate 1 scheme, Fig. 2a).

Additionally, the evenness of distribution of colistin in agar was evaluated by plating the same strain inoculum in a minimum of five different zones on the same colistin-containing plate. Finally, the impact of one-week storage of prepared colistin agar plates on colistin MICs was also tested using CS and CR strains.

All investigated methods was performed according to the CLSI guidelines [16]. For all experiments colistin sulfate salt was used (Lot#SLBD8306V or SLBQ0243V, Sigma-Aldrich, St. Louis, USA). All tubes and plates were incubated in ambient air for 18 h at 35 ± 2 °C followed by visual assessment of turbidity or growth independently by two investigators in order to assess inter-investigator variability. Detailed methods are described in specific sub-sections below.

#### Agar dilution

**Fig. 1:**
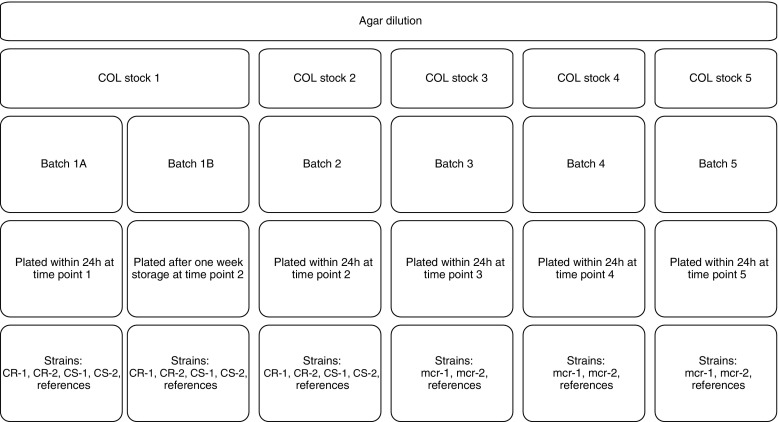
Agar dilution study design

**Fig. 2:**
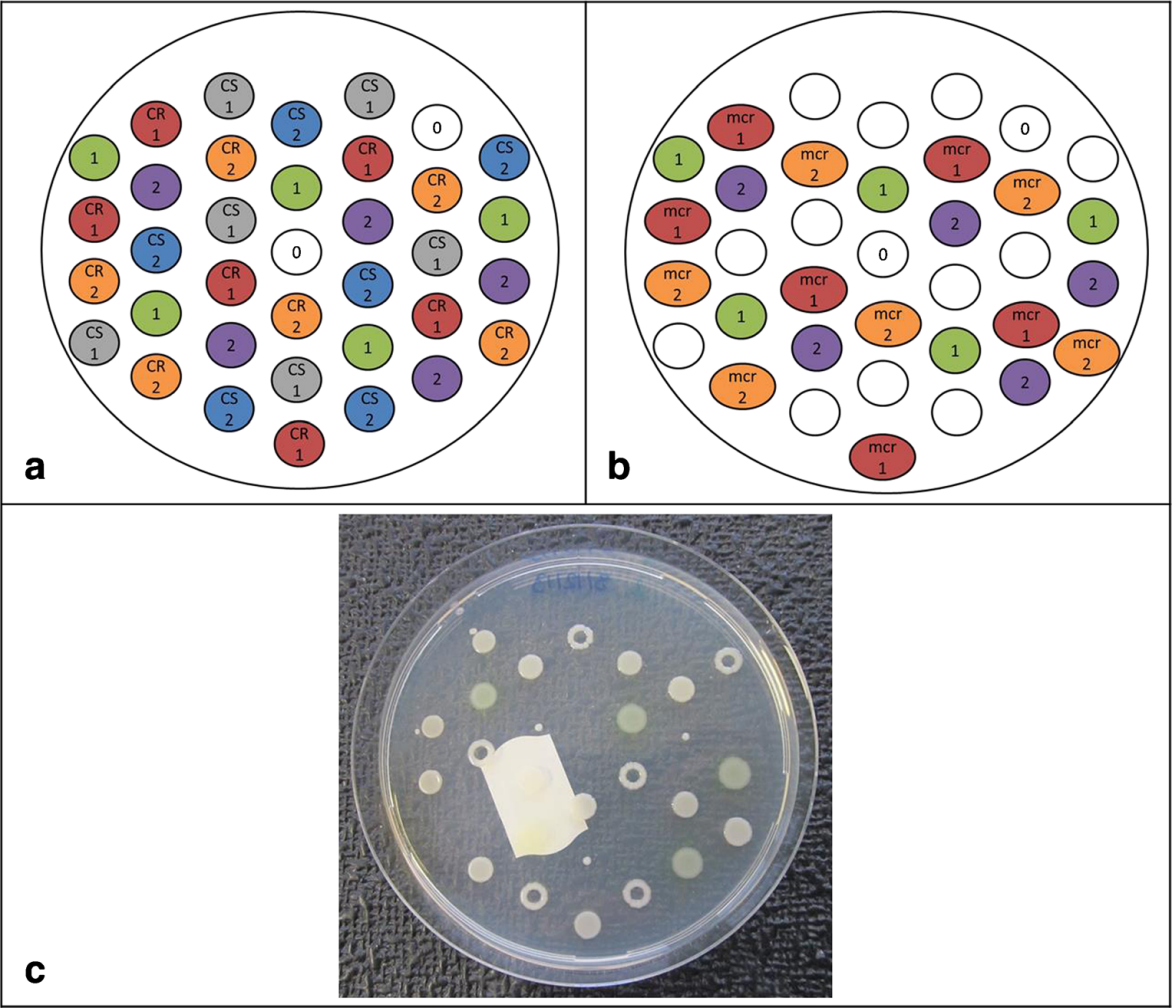
Strain distribution scheme (1 – ATCC 25922, 2 – ATCC 27853, 0 – blank) for agar dilution (plate 1 – **a**, plate 2 – **b**) and an example plate with test strains. Note the ring-shaped growth at certain spots (**c**)

Mueller-Hinton agar (MHA, BD Diagnostics, Le Pont de Claix, France) containing 0.125–256 μg/ml colistin was prepared in 90-mm plates in triplicate. A 0.5 McF suspension was diluted 1:10 of which 2 μl was inoculated on the prepared plates using a Multipoint Elite™ plater (Mast Group Ltd., Bootle, UK) resulting in a final bacterial inoculum of 1 × 10^4^ CFU/spot. In order to check for the solubility and distribution of colistin in agar plates, strains were spotted on different regions of the plate. Each clinical strain and the two reference strains were spotted on 6 and 5 different locations, respectively (Fig. 2a and b). Agar plate batches for CS and CR strains (plate 1) were prepared as follows: batch 1 was prepared on the same day, and half of the plates from this batch (1A) were inoculated immediately (within 24 h of media preparation) and the other half (1B) after 1 week of storage at 4 °C. Batch 2 was prepared 1 week later and freshly inoculated on the same day as batch 1B to enable comparison between MICs observed on fresh and stored plates using the same strain inoculum (Fig. 1). *Mcr*-harboring strains were tested independently of the CS and CR strains in triplicate on freshly prepared agar plates that were inoculated within 24 h (plate 2 scheme, Fig. 2b). Plating for each batch was performed in triplicate and from the three batches plated we obtained readings from a total of nine plates corresponding to 54 spots for clinical and 45 spots for reference strains.

#### Broth dilution

Bacterial inoculum of 5 × 10^5^ CFU/ml in cation-adjusted Mueller-Hinton broth (CAMHB, BD Diagnostics, Le Pont de Claix, France) was used for broth MIC testing as described in CLSI guidelines [17]. Briefly, serial two-fold dilutions were prepared in CAMHB ranging from 0.125 to 256 μg/ml colistin concentrations, and 1 ml of each dilution was distributed in glass tubes for broth macrodilution. For broth microdilution, 0.05 ml of each dilution was distributed over a 96-well polystyrene microwell-plate (CELLSTAR®, Greiner Bio-One GmbH, Frickenhausen, Germany). Additionally, given the known problems of colistin binding to plastic/polystyrene [5], we also explored the utility of glass-bottomed microwell plates (Nunc™ Lab-Tek™ 16 cells Chamber Slide, Thermo Fisher Scientific, Rochester, USA) for broth microdilution testing. These were similarly inoculated as the polystyrene microwell plates.

#### MIC gradient strips

A 0.5 McF suspension was spread on Mueller-Hinton agar (BD Diagnostics, Le Pont de Claix, France), and colistin MIC gradient strip was applied. We used colistin Etest® strip (bioMérieux SA, Marcy l’Etoile, France) for CR and CS strains testing; however, due to non-availability, we utilized Liofilchem® MIC Test Strip (MTS, Liofilchem s.r.l., Roseto degli Abruzzi, Italy) for the *mcr* strains. Also when testing *mcr* positive strains, only ATCC 25922 was used as a reference.

#### Quality control

To confirm the initial inoculum size, 100 μl of 10^-5^ and 10^-6^ dilution of the 0.5McF was spiral-plated (EddyJet, IUL SA, Barcelona, Spain) on MHA. Colonies were counted manually using a Stuart™ Scientific colony counter SC5 (BIBBY Sterilin Ltd., Stone, UK).

### Heteroresistance assay

One colistin-susceptible strain, CS-1, was subjected to 2X, 4X, and 8X its colistin MIC (1 μg /ml) to observe emergence of a heteroresistant subpopulation. Briefly, colistin dilutions of 2, 4, 6 and 8 μg/ml were prepared in CAMHB. 20 μl of the colistin stock dilutions were distributed in a 96-well polystyrene plate (CELLSTAR®), and 160 μl of CAMHB was added. A 0.5McF suspension (≈1.5 × 10^8^ CFU/ml) was prepared from a fresh overnight culture of CS-1, and 20 μl was added to each microwell in the 96-well plate in triplicate. Growth was monitored every 15 min for 24 h at 37 °C using a Multiskan™ GO Microplate Spectrophotometer (Thermo Fisher Scientific, Vantaa, Finland). Validation of resistant subpopulations was performed by plating on MHA plates containing either 0 μg/ml or 8 μg/ml of colistin using an Eddy Jet spiral plater followed by overnight incubation at 37 °C and subsequent broth macrodilution MIC testing.

### Statistical analysis

Means and standard deviations were calculated and graphs were generated using IBM SPSS Statistics 24. Mean log_2_ values of the MIC were calculated for graphical representation. Cohen’s kappa coefficient was calculated to investigate the agreement level between the two investigators reading the MIC results.

## Results

In this study, along with comparing known methods of colistin MIC testing and their reproducibility, we also assessed the stability of colistin in agar plates and the inter-investigator variability in estimating colistin MICs. For the latter, level of agreement between the readout of two independent investigators was found to be excellent (Cohen’s kappa coefficient 0.948). Therefore, readings from only one investigator were considered for further analysis.

### Agar dilution

#### Colistin distribution in agar and intra-plate reproducibility

**Fig. 3:**
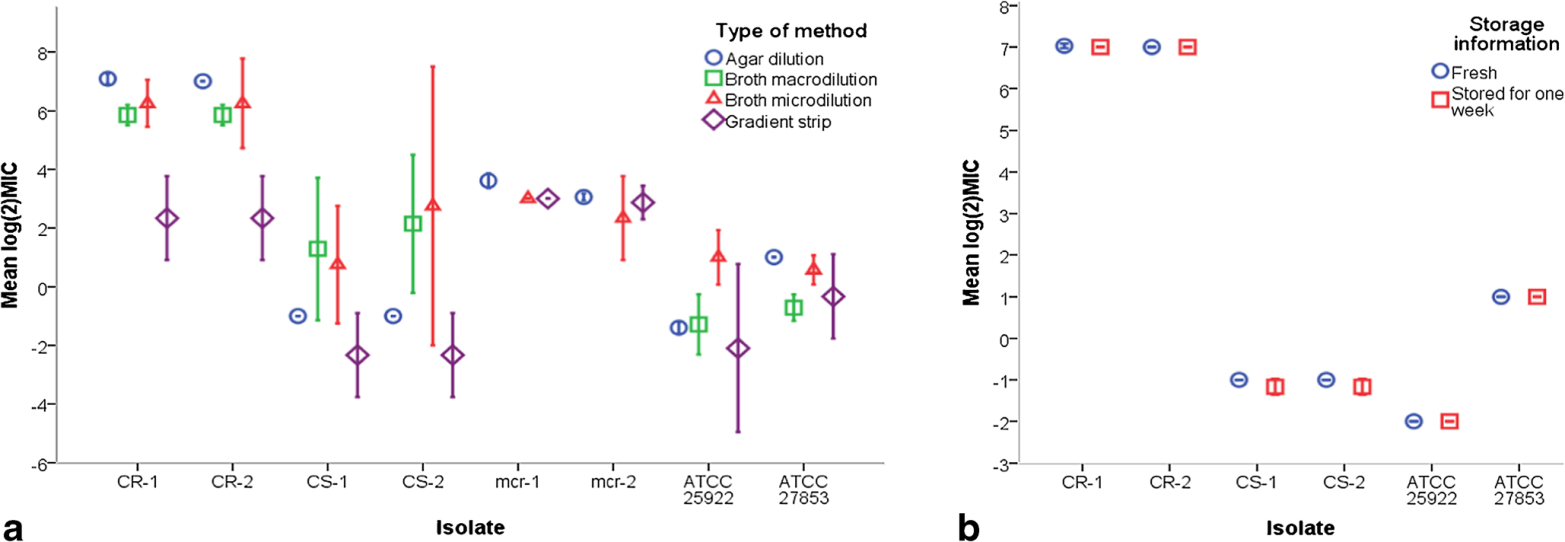
Overview of the mean values of log_2_MICs of all the tested methods (**a**) and influence of one-week agar plate storage on log_2_MIC value (**b**). Error bars represent 95% confidence interval

Agar dilution MIC results were analyzed at various levels. First, the evenness of colistin distribution and intra-plate reproducibility was assessed by spotting the same strain inoculum at six different spots on the same plate for clinical isolates and five distinct spots for reference ATCC strains (Fig. 2a and b). Each MIC run was performed in triplicate resulting in 18 readouts for each clinical strain and 15 for ATCC strains. Comparison of the two fresh batch MIC runs (1A and 2) showed that all spots of both CR-1 and CR-2 grew at 64 μg/ml colistin concentrations (MIC 128 μg/ml); one spot of CR-1 also grew at 128 μg/ml colistin (MIC 256 μg/ml). For the CS-1 and CS-2 strains, all spots from both runs grew uniformly at 0.25 μg /ml concentrations (MIC 0.5 μg/ml) (Fig. 3a). The triplicate MIC testing of the *mcr*-harboring strains showed an MIC of 16 μg/ml for the *mcr-1* strain in two batches with exception of one spot in one batch that showed MIC of 8 μg/ml. In the third batch testing, all spots of the *mcr-1* strain showed an MIC of 8 μg/ml. Results of the *mcr-2* positive strain showed the MIC to be 8 μg/ml for all three batches in all spots but one. This one spot had an MIC of 16 μg/ml (Fig. 3a).

In summary, technical replicates of both colistin-susceptible and -resistant, as well as *mcr* harboring clinical isolates showed reproducible MICs both within the plate and between the two runs. Intra- and interplate MICs of control strains, ATCC 25922 and ATCC 27853, were constant and within range at 0.25 μg/ml and 2 μg/ml, respectively.

#### Impact of one-week storage of colistin containing agar plates on MICs

No differences were found when comparing MICs of the two ATCC control strains obtained from the 1-week-old colistin agar plates and from the freshly used plates. On all spots on all plates the MIC values were 0.25 μg/ml and 2 μg/ml for ATCC 25922 and ATCC 27853, respectively. Results for CR strains on the 1-week stored plates were also uniform and showed MICs of 128 μg/ml. For the CS strains, the overall MIC on stored plates was also 0.5 μg /ml, as for the fresh batches, although a one dilution lower MIC was observed for both strains in a few spots. MICs of 0.25 μg/ml were observed for CS-1 (one spot on all three replicate plates) and for CS-2 (single spot on the stored plates and replicate 2, and two spots on replicate 3 plates) (Fig. 3b).

Together this data shows that there is no difference in MIC values depending on the region of plate where the tested strain was spotted. This indicated that colistin distribution in agar was uniform and that agar dilution is a reliable and reproducible method for colistin MIC determination, even when plates are stored for 1 week at 4 °C.

We also observed an interesting phenomenon with our colistin-susceptible strains, that to our knowledge has not been reported before, which is a ring-shaped growth of bacteria in the inoculated spot on agar plates (Fig. 2c). If present, this was consistently observed at colistin concentrations one dilution lower than the strain’s MIC.

### Broth macrodilution

The MICs of CS and CR strains were tested in seven independent experiments with broth macrodilution and the overview of obtained results is presented in Fig. 3a. For ATCC 25922, median MICs was 0.25 μg/ml and ranged between 0.25–2 μg/ml. Whereas for ATCC 27853, median MICs was 0.5 μg/ml (0.5–1 μg/ml). Median MIC values for CR strains were 64 μg/ml (range 32–64 μg/ml), while MIC values for the CS strains were more diverse ranging from 0.25 to 32 μg/ml for CS-1 with median MIC of 2.25 μg/ml and from 0.5 to 32 μg/ml for CS-2 with median MIC of also 2.25 μg/ml.

Of note, ‘skipped’ wells (lack of growth) [15] were observed for both CS-1 and CS-2 in several of these runs. For CS-1, in one run no growth was observed at 2 μg/ml colistin, but was observed at 4 μg/ml; in another run multiple skips were observed in colistin concentrations 0.125–1 μg/ml, making this run uninterpretable. Similar skips were observed for CS-2 in three runs: no growth at 0.25 μg/ml in one run that gave MIC 1 μg/ml, no growth at 1 μg/ml in a run that gave MIC 16 μg/ml, and finally no growth from 0.5–8 μg/ml in the third run that gave MIC of 32 μg/ml, making the last run uninterpretable.

### Broth microdilution

Broth microdilution was performed in 96-well polystyrene plates 3–4 times for all strains, and additionally, twice in polystyrene plates with glass bottom for the CS and CR strains. Results obtained in 96-well polystyrene plates are summarized in Fig. 3a. For ATCC 25922, median MIC was 2 μg/ml (0.5–4 μg/ml), whereas for ATCC 27853, it was 2 μg/ml (1–2 μg/ml). CS-1 had median MIC of 2 μg/ml ranging from 0.5 to 4 μg/ml, and CS-2 median MIC was 2.25 μg/ml and ranged from 0.5 to 4 μg/ml. Two runs of MIC testing for CS-2 were uninterpretable due to ‘skipped’ wells. CR-1 and CR-2 median MICs were 64 μg/ml (64–128 μg/ml) and 96 μg/ml (32–128 μg/ml), respectively. As for *mcr-1* on all three repeats the MIC was 8 μg/ml, and for *mcr-2* the median MIC was 4 μg/ml (4–8 μg/ml).

In the broth microdilution assay in polystyrene plates with a glass bottom, ATCC 25922 and ATCC 27853 showed MICs of 0.25 μg/ml and 0.5 μg/ml, respectively, for both runs. For CS-1, MICs were 0.5 and 16 μg/ml; for CS-2, 0.25 μg/ml and uninterpretable (growth until 2 μg/ml, but skips at 0.25 and 1 μg/ml); for CR-1, 64 and 64 μg/ml; and for CR-2, 32 and 32 μg/ml, in the two runs.

### MIC gradient strips

Colistin-containing MIC gradient strips were tested in triplicate and results are shown in Fig. 3a. The median MICs for both CR-1 and CR-2 was 4 μg/ml and ranged from 4 to 8 μg/ml. For CS-1 and CS-2, MICs ranged from 0.125–0.25 μg/ml for both strains, with median MIC of 0.25 μg/ml for each strain. For ATCC 27853, the median MIC was 1 μg/ml (0.5–1 μg/ml), while for ATCC 25922 the MICs 0.125 μg/ml were observed in all three runs with Etest, whereas with MTS it was 1.5 μg/ml. The *mcr-1* strain had MIC of 8 μg/ml in all three runs, whereas *mcr-2* strain had median MIC of 8 μg/ml (6–8 μg/ml).

### Heteroresistance assay

**Fig. 4:**
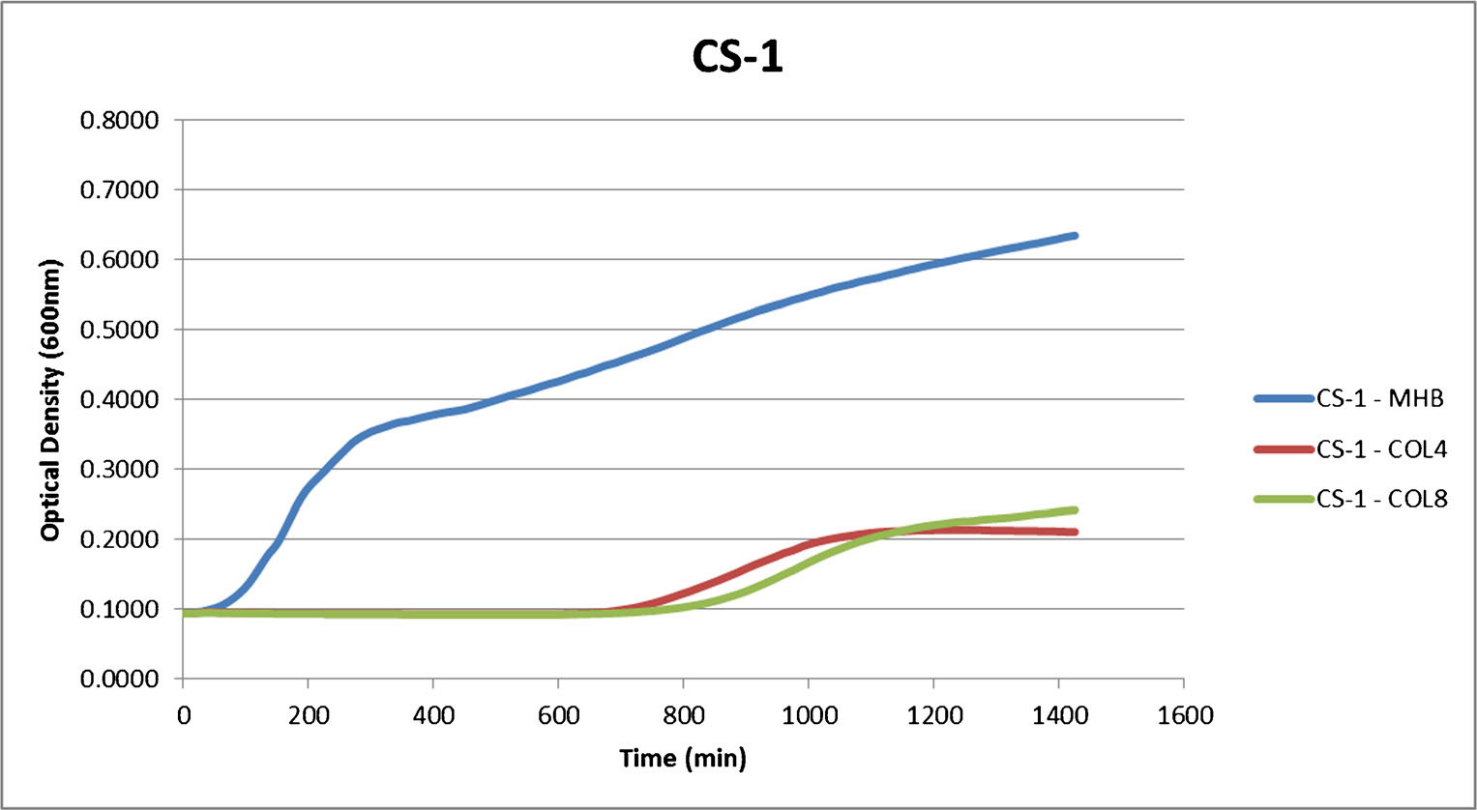
Extended growth assays utilizing the CS-1 *K. pneumoniae* without colistin (MHB) and under various colistin concentrations

Despite CS-1 being colistin-susceptible, turbidometric measurements showed growth in colistin concentrations >2 μg/ml that were observable after ~700 min of incubation (Fig. 4). As growth might have resulted from the strain becoming resistant or because colistin was hydrolysed, we tested 100 μl of a control strain (*E. coli* ATCC 25922) and of the “resistant” subpopulation of CS-1 on plates containing colistin 4, 6 and 8 μg/ml in order to exclude the latter possibility. While no colonies were observed for the control strain, the “resistant” subpopulation of CS-1 yielded high numbers of colonies that showed MICs to colistin ranging from 4 to 8 μg/ml.

## Discussion

As yet, there is no consensus on methodology for colistin susceptibility testing [16, 18]. The method most commonly used in routine laboratories, disc diffusion, has been shown to be unreliable due to poor colistin diffusion in agar [7–9]. Therefore, MIC testing is suggested instead, but it is not clear which method is optimal [9, 11, 16, 18]. Recently, the joint CLSI–EUCAST Polymyxin Breakpoints Working Group recommended broth microdilution in non-treated polystyrene trays using cation-adjusted MHB without any additives as a reference method (http://www.eucast.org/ast_of_bacteria/guidance_documents). As for other MIC methods, in the same document it was mentioned that at the moment agar diffusion MIC methods and agar dilution are not recommended and urged the need of further validation with new study data. Therefore, we decided to compare different colistin MIC methods: agar dilution, broth dilution (micro- and macro-) and MIC gradient strips, using two reference strains with well-defined colistin MIC ranges, as well as four clinical isolates.

The greatest challenge in colistin handling is its binding to plastic [19]. Because of this property, we decided to limit as much as possible the contact of colistin with plastic. Antibiotic stock and dilutions were prepared in glass bottles. We did not exclude the use of automatic pipettes with plastic tips, since this is a very handy and standard way of measuring the desired volumes. Plastic pipettes were also utilized for pouring the agar plates. The solubility, stability and distribution of colistin in agar plates were investigated by testing the same bacterial strain suspension in different regions of the agar plate and comparing the obtained MIC values. Additionally, the impact of one-week storage of colistin containing plates at 4 °C on MICs was tested. This is a practical issue as bulk production of MIC plates is commonly practiced in microbiology laboratories for increased cost-effectiveness and efficiency.

MIC values for all strains tested with agar dilution were highly reproducible, not only among spots on the same plate, but also among replicates of fresh plates and stored ones. In contrast to the other methods tested, no difference was observed in reproducibility of MIC determination by agar dilution for both colistin-susceptible and resistant strains. This confirms not only equal distribution of colistin in the agar plates and reliability of this method for MIC determination, but also a good stability of antibiotic in MH agar under the proper storage conditions. Agar dilution has been shown to be reliable for colistin MIC determination in several studies [11, 20, 21] and was also successfully utilized for screening purposes [22].

The MIC values obtained by agar dilution were compared to those obtained from broth dilution and MIC gradient strips. MICs for the two reference strains were in the acceptable range (0.25–2 and 0.5–4 μg /ml for ATCC 25922 and ATCC 27853, respectively) for all tested methods except Etest and broth microdilution, where MIC of ATCC 25922 was one dilution lower (0.125 μg/ml) and one dilution higher (4 μg/ml), respectively. Differences in MICs of the CR strains obtained with Etest were remarkably lower (4–8 μg/ml) than those obtained by the other methods (32–128 μg/ml). Although it did not result in misinterpretations of colistin-resistant strains as susceptible, this might be because both strains utilized here were high-level resistant. It could thus be hypothesized that if lower-level colistin-resistant strains had been utilized, these might have been detected as susceptible [12]. However, when the *mcr* harboring strains with lower resistance levels were tested with gradient strips, the MIC results were similar to other methods. The performance of the colistin gradient diffusion methods has been questioned in some studies [5, 12, 21], but seemed to be reliable in others [7, 8, 10], which may be related to the MIC ranges of the strains tested and the underlying colistin resistance mechanism.

We found that broth dilution methods showed remarkably high deviations, especially in case of colistin-susceptible clinical strains wherein MICs shifted from sensitive to resistant in a few runs. Such wide MIC ranges have been commonly linked to ‘skipped’ wells, a phenomenon that has been reported previously for *A. baumannii*, *P. aeruginosa* and *E. cloacae* [23–25], and is primarily attributed to heteroresistance. Recently, it has also been described in *K. pneumonia* isolates [26]. Here, we demonstrate the ease with which colistin-resistant subpopulations either arise or are amplified from a primarily colistin-sensitive strain under colistin pressure. To further validate our classification of these strains as colistin sensitive, we whole genome sequenced CS-1 and did not find any mutations or other changes previously associated with colistin resistance (data not shown). Of note, the phenomenon of skipped wells was not observed for *mcr* harboring strains, where colistin resistance is mediated by a plasmid-acquired phosphoethanolamine and is not due to mutations/changes in chromosomal genes. This is expected as heteroresistance refers to the presence, within a larger population of fully antimicrobial-susceptible microorganisms, of subpopulations with lesser susceptibility. Importantly, to understand the differences in frequency of emergent heteroresistant populations between agar dilution and broth dilution, it is important to consider the basic differences in methodologies, i.e., the cation-adjustment of broth and the different bacterial loads used for inoculation as recommended by CLSI guidelines [17]. In case of agar dilution, the total inoculum spotted is 10^4^ CFU, whereas for broth dilution it is higher, 5 × 10^5^ CFU. While a larger isolate panel would be required to make definitive conclusions, our data do indicate that use of higher bacterial loads for MIC testing might be enriching for resistant sub-populations in colistin-sensitive clinical isolates.

To further investigate the influence of binding of colistin to polystyrene on MIC testing [19], we compared broth microdilution results obtained in standard 96-wells plate to results from plates with glass bottom. For reference and for CR strains the results were more reproducible than with the standard broth microdilution method. The MICs for ATCC strains were one dilution lower than the lowest obtained in 96-well plates, whereas for the CR strains the MICs were the same as the lowest in typical plates. This trend was, however, not confirmed for CS strains, which might be linked to heteroresistance.

One of the weaknesses of our study was the use of media from a single brand (MHA and CAMHB provider) as this has been shown to influence results. However, despite this and the use of a small panel of strains, our experiments were iterated several times and were well-controlled. Hence, these could form the basis for testing a larger number of strains across methods to define a suitable and robust method for colistin susceptibility testing.

In conclusion, we found the agar dilution method to be superior in terms of reproducibility, robustness and ease of use compared to the currently recommended broth dilution methods tested here for colistin MIC determination. These observations may justify more extensive validations of agar dilution, with the goal of developing a globally accepted standardized protocol for colistin susceptibility testing.

## Abbreviations

CFU: Colony forming units
MIC: Minimal inhibitory concentration
